# Rapid and accurate pyrosequencing of angiosperm plastid genomes

**DOI:** 10.1186/1471-2229-6-17

**Published:** 2006-08-25

**Authors:** Michael J Moore, Amit Dhingra, Pamela S Soltis, Regina Shaw, William G Farmerie, Kevin M Folta, Douglas E Soltis

**Affiliations:** 1 Department of Botany, University of Florida, P.O. Box 118526, Gainesville, FL, 32611, USA; 2 Florida Museum of Natural History, University of Florida, P.O. Box 117800, Gainesville, FL, 32611, USA; 3 Horticultural Sciences Department, University of Florida, P.O. Box 110690, Gainesville, FL, 32611, USA; 4 ICBR Genome Sequencing Service Laboratory, University of Florida, P.O. Box 100156, Gainesville, FL, 32610, USA

## Abstract

**Background:**

Plastid genome sequence information is vital to several disciplines in plant biology, including phylogenetics and molecular biology. The past five years have witnessed a dramatic increase in the number of completely sequenced plastid genomes, fuelled largely by advances in conventional Sanger sequencing technology. Here we report a further significant reduction in time and cost for plastid genome sequencing through the successful use of a newly available pyrosequencing platform, the Genome Sequencer 20 (GS 20) System (454 Life Sciences Corporation), to rapidly and accurately sequence the whole plastid genomes of the basal eudicot angiosperms *Nandina domestica *(Berberidaceae) and *Platanus occidentalis *(Platanaceae).

**Results:**

More than 99.75% of each plastid genome was simultaneously obtained during two GS 20 sequence runs, to an average depth of coverage of 24.6× in *Nandina *and 17.3× in *Platanus*. The *Nandina *and *Platanus *plastid genomes shared essentially identical gene complements and possessed the typical angiosperm plastid structure and gene arrangement. To assess the accuracy of the GS 20 sequence, over 45 kilobases of sequence were generated for each genome using conventional sequencing. Overall error rates of 0.043% and 0.031% were observed in GS 20 sequence for *Nandina *and *Platanus*, respectively. More than 97% of all observed errors were associated with homopolymer runs, with ~60% of all errors associated with homopolymer runs of 5 or more nucleotides and ~50% of all errors associated with regions of extensive homopolymer runs. No substitution errors were present in either genome. Error rates were generally higher in the single-copy and noncoding regions of both plastid genomes relative to the inverted repeat and coding regions.

**Conclusion:**

Highly accurate and essentially complete sequence information was obtained for the *Nandina *and *Platanus *plastid genomes using the GS 20 System. More importantly, the high accuracy observed in the GS 20 plastid genome sequence was generated for a significant reduction in time and cost over traditional shotgun-based genome sequencing techniques, although with approximately half the coverage of previously reported GS 20 *de novo *genome sequence. The GS 20 should be broadly applicable to angiosperm plastid genome sequencing, and therefore promises to expand the scale of plant genetic and phylogenetic research dramatically.

## Background

Plastid genome sequence information is of central importance to several fields of plant biology, including phylogenetics, molecular biology and evolution, and plastid genetic engineering [1-6]. The relatively small size of the plastid genome (~150 kb) has made its complete sequencing technically feasible since the mid-1980s, although limitations in sequencing technology resulted in only a few complete plastid genomes appearing between 1986 and 2000 [7]. However, the pace of plastid genome sequencing has increased markedly over the last five years [7]. More than 50 complete plastid genomes are now available on GenBank, and several plastid genome sequencing projects [8-10] promise to increase that number to more than 200 in the near future. This dramatic growth in plastid genome sequencing has been driven largely by improvements in Sanger sequencing technology that have greatly reduced the time and cost involved in genome sequencing [11].

New approaches to genome sequencing have been proposed in recent years that, if effective, will further significantly reduce the time and cost of obtaining whole plastid genome sequences [11,12]. Perhaps the most promising of these new technologies involves the Genome Sequencer 20 (GS 20) System, a pyrosequencing platform developed by the 454 Life Sciences Corporation (Branford, CT, USA; available through Roche Diagnostics, Indianapolis, IN, USA). In pyrosequencing, the DNA sequence is determined by analyzing flashes of light that are released during the enzymatic conversion of pyrophosphate generated during template DNA extension, using a predetermined sequence of dNTP addition [13]. The GS 20 System implements several novel technologies that allow for relatively rapid and inexpensive pyrosequencing on a massive scale [14]. These include an emulsion-based method to amplify random fragment libraries of template DNA in bulk, fiber-optic slides containing high-density, picoliter-sized pyrosequencing reactors, and a three-bead system to deliver the enzymes necessary for the pyrosequencing reactions. In a single run the GS 20 system generates up to 25 million high-quality bases in hundreds of thousands of short sequence reads called flowgrams, which are then assembled into genomic contigs. For relatively small genomes, the high number of reads results in a high average depth of sequence coverage, effectively overcoming many of the limitations of pyrosequencing, which include relatively short read length and uncertainty in the length of homopolymer runs [14,15]. Perhaps the greatest advantage of the GS 20 System is that it generates genome sequence much more rapidly and economically than traditional Sanger-based shotgun sequencing. It is not necessary to clone template DNA into bacterial vectors, and genome sequence can be obtained on the GS 20 in a single five-hour run with a few days of template preparation. Likewise, the GS 20 System relies on less expensive reagents than traditional Sanger sequencing. However, the savings in time and money associated with GS 20 *de novo *genome sequence comes at the cost of a slightly higher error rate compared to traditional Sanger-based genome sequence (~0.04% in GS 20 vs. 0.01% in Sanger sequence) [14,16,17].

To date the GS 20 System has been successfully utilized in an increasing number of *de novo *sequencing projects, including sequencing the genomes of several bacteria and the mitochondrial genome of an extinct species of mammoth, as well as exploring the sequence diversity present in environmental samples [14,18-22]. Because of its small size and similarity to bacterial genomes, the plastid genome seems particularly amenable to sequencing via the GS 20 System. In conjunction with the Angiosperm Tree of Life (ATOL) project [8], part of which involves sequencing 30 plastid genomes representing the phylogenetic diversity of angiosperms, we used the GS 20 to sequence the complete plastid genomes of the eudicot angiosperms *Nandina domestica *Thunb. (Berberidaceae) and *Platanus occidentalis *L. (Platanaceae). A major focus of the ATOL plastid genome sequencing project is the use of whole-chloroplast genome sequence data to determine the evolutionary relationships among the basal lineages of eudicots, which have hitherto proved difficult to resolve [23]. We therefore sequenced *Nandina *and *Platanus *because they represent members of two phylogenetically pivotal basal lineages of eudicots (Ranunculales and Proteales, respectively), which shared their last common ancestor approximately 120 million years ago [24]. In sequencing these two plastid genomes using the GS 20 System we had the following specific objectives: (1) to test the overall feasibility of generating plastid genome sequence using the GS 20 System, (2) to determine the potential error rate in GS 20 *de novo *plastid genome sequence, and (3) to determine whether the magnitude of the GS 20 error rate is enough to offset any potential gains in time and cost efficiency associated with the use of the GS 20. Here we demonstrate the viability of the GS 20 System for plastid genome sequencing projects by generating highly accurate and essentially complete plastid genome sequences of both *Nandina *and *Platanus*, for a significant reduction in time and cost over traditional Sanger-based plastid genome sequencing.

## Results

### GS 20 sequencing run characteristics

Results of the GS 20 sequencing runs for *Nandina *and *Platanus *are summarized in Table 1. More than 99.75% coverage of each genome was obtained by assembling the raw sequence data from the titration and supplemental sequencing runs (these data will be referred to as the combined run data; see Methods), to an overall average depth of coverage of 24.6× in *Nandina *and 17.3× in *Platanus*. Few gaps were present in either genome assembly (Table 1). All but three gaps were less than 50 bp, with many zero-length gaps (no missing sequence between adjoining contigs) present in both assemblies. Only one gap in either assembly was larger than 100 bp (in *Platanus*; Table 1). In several cases gaps in the assemblies occurred in the same regions of both genomes. Short gaps (mostly zero-length, but all < 5 bp) were present at all four junctions between the inverted repeat (IR) and single-copy (SC) regions in both *Nandina *and *Platanus*, as well as within the *rpoB *gene (32 bp and 27 bp gaps, respectively) of each genome.

### Genome characteristics

**Figure 1:**
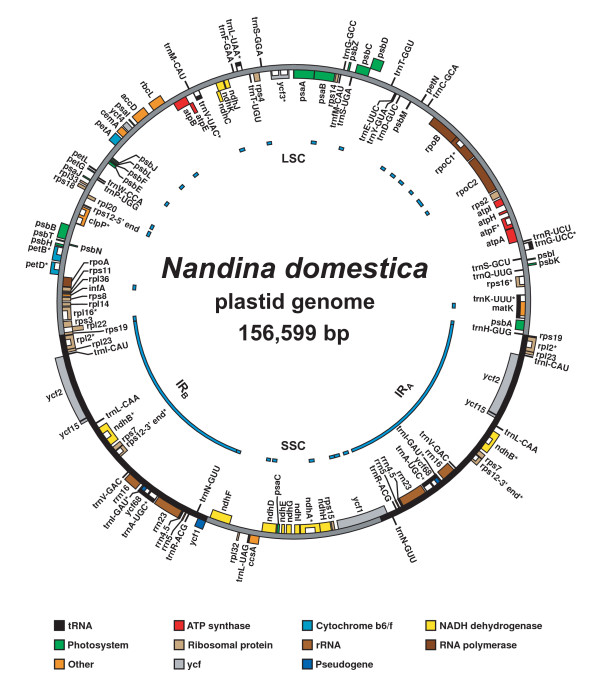
**Plastid genome map of *Nandina domestica *(Berberidaceae)**. Map of the plastid genome of *Nandina domestica *(Berberidaceae), showing annotated genes and introns. Asterisks (*) after the gene names indicate the presence of introns; the introns themselves are denoted by white boxes within genes. Within the genome map, the inverted repeat regions (IR_A _and IR_B_) are depicted by the solid black bars, and the large and small single-copy regions (LSC and SSC) are depicted by the solid gray bars. Regions that were conventionally sequenced are indicated by the blue bars to the inside of the genome map.

**Figure 2:**
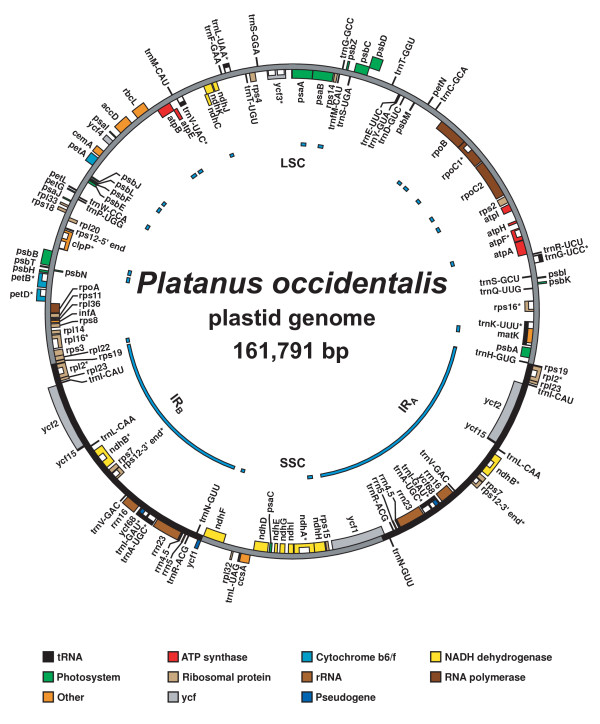
**Plastid genome map of *Platanus occidentalis *(Platanaceae)**. Map of the plastid genome of *Platanus occidentalis *(Platanaceae), showing annotated genes and introns. Asterisks (*) after the gene names indicate the presence of introns; the introns themselves are denoted by white boxes within genes. Within the genome map, the inverted repeat regions (IR_A _and IR_B_) are depicted by the solid black bars, and the large and small single-copy regions (LSC and SSC) are depicted by the solid gray bars. Regions that were conventionally sequenced are indicated by the blue bars to the inside of the genome map.

The plastid genomes of both *Nandina *and *Platanus *possess the typical genome structure observed in most angiosperm plastids, with an IR region of ~25 kb separating large and small SC regions (Figs. 1, 2; Table 2) [25,26]. Neither genome is rearranged relative to *Nicotiana *[27,28]. The plastid genomes of *Nandina *and *Platanus *share essentially identical complements of coding genes, each containing 30 tRNA genes, 4 rRNA genes, and 79 protein-coding genes (Table 3). Based on the presence of internal stop codons, two pseudogenes (*ycf15 *and *ycf68*) are present in the *Platanus *plastid genome. In *Nandina *the latter locus is also present as a pseudogene, although *ycf15 *appears intact. Both of these genes have been frequently reported as pseudogenes in other angiosperms [29,30], and so their presence as pseudogenes in *Nandina *and *Platanus *is not surprising. Based on the presence of ACG start codons in their DNA sequence, RNA editing appears to be necessary for the proper translation of two genes in *Nandina *(*ndhD *and *rpl2*) and three genes in *Platanus *(*ndhD*, *psbL*, and *rpl2*), and likely occurs throughout each genome on a broader scale [28,31].

### Accuracy of the GS 20 sequence

Conventional sequencing of the IR, IR/SC junctions, and regions surrounding putative coding sequence errors resulted in 46134 bp of comparison sequence in *Nandina *and 45249 bp of comparison sequence in *Platanus*. Observed error rates in the combined run data for these regions are summarized in Table 4. Observed numbers of errors in combined run data and lengths of conventional sequence data that were used in the error calculations are presented in Table 5. The overall observed error rate was 0.043% in *Nandina *and 0.031% in *Platanus*, and the combined overall error rate for both genomes was 0.037% (Table 4).

**Figure 3:**
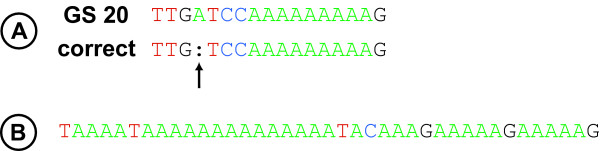
**Illustrations of a transposition-like insertion error and a homopolymer run set**. Illustrations of a transposition-like insertion error and a homopolymer run set. (A) Comparison of a hypothetical stretch of GS 20 genome sequence (top) vs. the "correct" sequence (bottom) in order to illustrate an example of a transposition-like insertion error, in which a base identical in composition to a given HR is inserted in a nearby, nonadjacent position. The transposition-like insertion error in the GS 20 sequence is indicated by the arrow; the colon (:) in the "correct" sequence indicates the absence of the A at the same position. (B) Example of a homopolymer run set.

Two types of errors were observed in the GS 20 combined data sequence: errors associated with contig ends, and insertions and deletions (indels), usually associated with homopolymer runs. A small number of errors was present within 50 bp of the ends of the combined data contigs in both genomes (5 errors in *Nandina *and 6 errors in *Platanus*). Including these errors increased overall error rates to 0.054% in *Nandina *and 0.044% in *Platanus*. However, these errors were excluded from other error calculations because they were expected as a result of the low depth of coverage at contig ends, and because such errors were necessarily checked by targeted Sanger sequencing when bridging the gaps between contigs, unlike the remaining, higher-coverage regions of the GS 20 assembly. All remaining errors were indels, all but one of which (a C/G insertion in *Platanus*) were directly associated with homopolymer runs (HRs). All HR-associated indel errors fell into two overall classes (summarized in Table 6). Approximately 85% of all errors associated with HRs involved length variation in the number of bases in a given HR. The remaining HR-associated errors involved the insertion of a base identical in composition with a given HR to a nearby, nonadjacent position. Because these insertions appear similar to transpositions, they are referred to as transposition-like insertions. An illustration of a transposition-like insertion is provided in Figure 3A.

Substitution errors were not definitively observed in either genome, although two differences in base composition between the conventional and GS 20 sequence were observed in the IR of *Nandina*. However, because the conventional IR sequence for *Nandina *was derived from a separate individual than that used in the GS 20 sequencing, it is likely that both differences result from interindividual variation, especially given that both sites possessed high-quality phred scores (> 40) in the GS 20 sequence. These two putative substitutions were therefore not included in error calculations.

**Figure 4:**
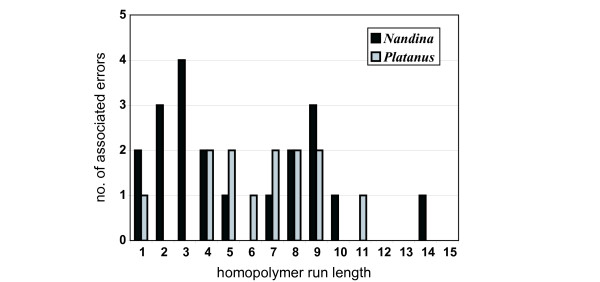
**Distribution of errors associated with homopolymer runs**. Distribution of errors associated with homopolymer runs, as a function of homopolymer run length.

Characteristics of the homopolymer runs associated with observed and estimated errors are also summarized in Table 6. More than 95% of all error-associated HRs in both genomes were A/T runs rather than C/G runs. A χ^2 ^test indicated that this A/T HR-associated error bias was significantly higher than would be expected given the observed A/T content of both genomes (*P *< 0.01 for both genomes). Approximately half of all errors occurred in regions characterized by groups of HRs of identical base composition interrupted occasionally by a differing base (these will be termed homopolymer run sets; an example is illustrated in Figure 3B). The length distribution of HRs associated with the observed errors is shown in Figure 4. Approximately 60% of all errors were associated with runs of 5 nucleotides or greater in both genomes. Of those errors associated with runs less than 5 nucleotides, all were associated with homopolymer run sets in *Platanus*, as were 10 of 11 such errors in *Nandina*. All 10 of the HR set-associated errors in *Nandina *occurred in a single 100-bp extensive HR set within the *trnV*/*rps12 *spacer in the inverted repeat. HR-associated insertion errors occurred more frequently than deletion errors in both genomes (~5× more frequently in *Nandina *and ~2.5× more frequently in *Platanus*; Table 6).

Nearly all insertion errors in both genomes occurred at sites with low or very low GS 20 quality scores (Table 7). Approximately 81% of all insertion errors had GS 20 phred-equivalent quality scores < 20, and approximately 93% of insertion errors had quality scores ≤ 40. However, one insertion error in each genome occurred at a site with a quality score > 40 (Table 7).

Errors were not distributed uniformly throughout either plastid genome (Table 4). The combined error rate across both genomes was higher in the SC regions than in the IR regions (0.047% in the SC regions and 0.029% in the IR regions). Regions of putative noncoding sequence also exhibited a higher error rate (~2× higher) than regions of putative coding sequence across both genomes (henceforth, putative coding and noncoding sequence will be referred to simply as coding and noncoding sequence). Similarly, error rates for noncoding sequence partitioned into IR and SC regions were higher than for coding sequence when pooled across both genomes (Table 4). The lowest overall error rates for both genomes were observed in the IR coding regions while the highest overall error rates were observed in the IR and SC noncoding regions. In both genomes at least one relatively small region contained a disproportionately large percentage of the total errors. A region of approximately 100 bp in the *trnV/rps12 *spacer of the *Nandina *genome contained 11 errors (representing 55.0% of all observed errors) in association with an extensive homopolymer run set. Likewise, three errors were observed in the *ycf1 *gene in both genomes (representing 15.0% of all errors in *Nandina *and 21.5% of all errors in *Platanus*), and three errors were also present in *rpoB *of *Platanus*.

## Discussion

Using the GS 20 System, we generated highly accurate and essentially complete plastid genome sequences simultaneously for two angiosperms in a short period of time (~2 weeks, including chloroplast isolation and library preparation) and for a significant reduction in cost (~$4500 per genome, including all library preparation and sequence run costs) over traditional shotgun-based genome sequencing methods. This savings in time and cost derives largely from the relative ease of template preparation and the extremely high throughput of the GS 20 System, which avoids the use of bacterial vectors and multiple rounds of expensive dye terminator-based sequencing reactions, both of which are necessary and time-consuming (taking several weeks to complete) components of Sanger-based shotgun sequencing [32]. We estimate that the GS 20 System requires approximately half the amount of template preparation time (~16 hours) compared to traditional Sanger-based methods (~36 hours) for plastid genome sequencing. Moreover, plastid genome sequencing using the GS 20 can be accomplished with two 4-hour instrument runs, while obtaining plastid genomes with Sanger-based shotgun sequencing requires several capillary sequencer runs (using 384-well plates) per genome. The small size of the plastid genome further contributes to the savings accompanying the GS 20 by allowing for multiple genomes to be sequenced simultaneously. The recent release of larger GS 20 PicoTiterPlates with the capacity to sequence up to four plastid genomes at a time promises to drive down the cost of GS 20 plastid genome sequencing even more, to ~$3500 per genome.

It is important to note that the savings observed in GS 20 sequencing of *Nandina *and *Platanus *also resulted from the lower average coverage obtained for these two chloroplast genomes (~20×) compared to that reported by Margulies et al. [14] for *de novo *genome sequencing (~40×). A similar reduction in coverage using Sanger-based sequencing methods would also result in a significant cost savings, perhaps still with a slightly higher sequence accuracy compared to the GS 20 genome sequence. However, to take full advantage of the ability to reduce coverage in Sanger-based plastid genome sequencing would require the sequencing of pure plastid DNA, something that can only reliably be achieved at present by constructing whole-genome bacterial artificial chromosome (BAC) libraries and then strictly sequencing plastid DNA-containing clones. The method of isolating plastid DNA using sucrose-gradient based chloroplast isolation and RCA (see Methods) that is employed in most angiosperm plastid genome sequencing projects is significantly less expensive than the construction of BAC libraries, although approximately 10–40% of the resulting RCA product consists of non-plastid DNA [7]. This contamination penalty must be overcome in Sanger-based sequencing through the addition of extra sequencing capacity, thereby partially mitigating against the significant savings that could be accrued through reducing sequence coverage. The same contaminants also reduce overall plastid genome coverage in GS 20 sequencing runs, but this does not impede the recovery of essentially complete plastid genomes at high accuracy, as evidenced by the sequencing of the *Nandina *and *Platanus *genomes. Thus the GS 20 instrument seems a reasonable and cost-effective alternative to Sanger-based shotgun sequencing with respect to angiosperm plastid genomics.

The generation of GS 20 genome sequence comes at the price of a slightly higher error rate (~0.04%) in comparison to Sanger sequencing (~0.01%) [16,17]. Nevertheless, the small magnitude of this error is not enough to offset the potential gains in time and cost efficiency of the GS 20 system. It is possible that the addition of extra GS 20 sequencing lanes on the PicoTiterPlates could reduce error rates below that observed in *Nandina *and *Platanus*, particularly in regions of relatively lower coverage. However, adding more lanes for each genome would drive up the cost of sequencing by reducing the number of plastid genomes that could be sequenced per plate (currently, four plastid genomes per plate are possible with the recent release of larger PicoTiterPlates). Depending on the aims and fiscal resources of a given sequencing project, the extra cost imparted by additional PicoTiterPlate space may not outweigh the benefits of slightly lower error rates.

The quantitative and qualitative aspects of the observed error in the GS 20 genome sequence of *Nandina *and *Platanus *are similar to those reported in published GS 20 sequence data. Although the error rates in Margulies et al. [14] for *de novo *genome sequencing represent estimates derived from consensus quality scores rather than observed error rates derived from comparison to Sanger sequence, the overall error rate reported for bacterial genomes in [14] (0.04%) was similar to that observed in both plastid genomes (0.043% in *Nandina *and 0.031% in *Platanus*). Importantly, we achieved comparable error rates to Margulies et al. [14] at approximately half the coverage in [14]. This equivalent error rate of ~0.04% at lower coverage is the result of recent improvements in the GS 20 assembly software (version 1.0.52.06); assembling the *Nandina *and *Platanus *genomes using the older software resulted in much higher error rates for both genomes (0.07% for *Nandina *and 0.14% for *Platanus*). It is also interesting to note that the lower average coverage of *Platanus*, which resulted directly from the higher percentage of non-cpDNA contamination in the RCA product of *Platanus *(~44% contamination) vs. that of *Nandina *(~18% contamination), did not result in a higher error rate compared to *Nandina *(Table 4).

The high percentage of errors associated with HRs and HR sets in *Nandina *and *Platanus *is similar to that reported in previously published GS 20 genome sequence [14] and is unsurprising given the known limitations of pyrosequencing technology [15]. The relatively high percentage of errors associated with these long HRs or HR sets also imparted some of the nonuniformity observed in the distributions of errors in both genomes. Likewise, the higher frequency of such long homopolymer runs or sets in noncoding plastid regions [33] explains the higher observed error rates in noncoding regions of both genomes (Table 4). Finally, the A/T bias present in both genomes (Table 2) does not appear to be solely responsible for the high proportion of A/T-associated HR errors (Table 6). Whether this excess of A/T HR errors is a byproduct of the GS 20 pyrosequencing technology is difficult to determine without more extensive analyses of additional genome sequences.

Another primary factor influencing the nonrandom distribution of errors in both genomes was relative depth of coverage in a particular region. The lower error rates observed in the IR regions of *Platanus *probably resulted in part from the essentially double coverage of the IR vs. SC regions during GS 20 sequencing (although this relationship does not hold in *Nandina*; Table 1). It is also likely that the higher error rate observed in some areas of both plastid genomes, as for example in *ycf1 *and *rpoB*, resulted from lower GS 20 sequence coverage in these regions. The ultimate cause of this lower coverage is unknown, but a plausible explanation involves the relative underamplification of these regions during the RCA reactions [34].

As we have demonstrated, the presence of a small amount of error in GS 20 genome sequence is not a serious impediment to the future use of the GS 20 System. Because nearly all errors in GS 20 sequence involve HR-associated length variation, the few errors that occur in protein-coding sequence can be easily identified because they induce frameshifts. Such errors can then be corrected through conventional sequencing. The GS 20 System should therefore prove to be an extremely useful tool in generating sequence for plastid coding regions, with only minimal finishing required to achieve essentially 100% accuracy. The GS 20-derived noncoding sequence will also be highly accurate, although a small number of errors will remain in the unchecked noncoding regions. However, the great majority of these errors will be associated with long homopolymer runs or homopolymer run sets, which are regions that are known to evolve rapidly via length mutations [35,36]. Moreover, long homopolymer runs are also prone to PCR errors [37-39], and therefore even conventional sequencing cannot guarantee 100% accuracy in such regions. For these reasons short length variation in such areas is frequently removed from phylogenetic sequence alignments, and the few remaining unchecked errors in GS 20 sequence are therefore unlikely to cause major problems should they be included in phylogenetic analyses.

The GS 20 System thus appears to be a viable option for plastid genome sequencing projects, especially given that the strong conservation of gene content and order exhibited by the *Nandina *and *Platanus *plastid genomes is shared across the overwhelming majority of angiosperms [25,26]. Perhaps the only significant limitation to the current use of the GS 20 in angiosperm plastid genome sequencing is posed by highly rearranged plastid genomes. Such genomes are characterized by high numbers of repeats [26,40], which could drive misassemblies during GS 20 sequence analysis due to short GS 20 read lengths. However, because very few lineages of angiosperms contain highly rearranged plastid genomes (examples include the families Campanulaceae and Geraniaceae, as well as some legumes) [26], the GS 20 should prove widely applicable to most angiosperms, as well as land plants in general.

## Conclusion

The utility of the GS 20 has already been demonstrated in bacterial, mitochondrial, and environmental *de novo *sequencing projects [14,18-22], and it shows promise for a number of other high-throughput sequencing projects, including transcriptome sequencing and SNP discovery. Here we have successfully applied GS 20 pyrosequencing technology to sequence the entire plastid genomes of two distantly related angiosperms with a significant savings of time and cost over traditional shotgun-based sequencing methods. This savings was partially achieved by sequencing to a lower average coverage than that reported in other GS 20 *de novo *genome sequencing projects. Nevertheless, this ~20× level of coverage was sufficient for the near complete recovery of both plastid genomes with ~99.96% accuracy. The GS 20 System may well usher in a new era of rapid and inexpensive plastid genome sequencing, thereby revolutionizing the fields of plant genetics and phylogenetics by dramatically expanding the amount of sequence data available to both.

## Methods

Fresh leaf material of *Nandina *and *Platanus *was collected on the campus of the University of Florida for chloroplast isolation. Voucher specimens (*Nandina*, M. J. Moore 310; *Platanus*, M. J. Moore 309) have been deposited in the herbarium of the Florida Museum of Natural History (FLAS). Purified chloroplasts were isolated from approximately 8.2 g of *Nandina *leaf material and 30.8 g of *Platanus *leaf material, following the sucrose gradient protocol of Jansen et al. [7]. Two 25-mL sucrose step gradients were used for each species. The purified chloroplasts were lysed in a solution containing 1.0 μL chloroplasts, 4.0 μL 1× PBS, and 1.5 μL activated solution A [7]. The lysis reactions were incubated on ice for 10 min, and then were stopped using 3.5 μL of solution B [7]. The released chloroplast DNA (cpDNA) was amplified via rolling circle amplification (RCA) [41] using the Repli-G kit (Qiagen, Inc., Valencia, CA, USA), following the manufacturer's instructions. To assess the relative percentage of cpDNA vs. nuclear DNA, RCA products were digested with *EcoRI *and visualized on agarose gels following the protocol in Jansen et al. [7].

GS 20 library construction and sequencing were performed as described in the supplementary material and methods of Margulies et al. [14] with slight modifications as specified by 454 Life Sciences. Briefly, high molecular weight DNA from the RCA reactions was sheared by nebulization to a size range of 300–800 bp. DNA fragment ends were repaired and phosphorylated using T4 DNA polymerase and T4 polynucleotide kinase. Adaptor oligonucleotides "A" and "B" supplied with the 454 Life Sciences sequencing reagent kit were ligated to the DNA fragments using T4 DNA ligase. Purified DNA fragments were hybridized to DNA capture beads and clonally amplified by emulsion PCR (emPCR). DNA capture beads containing amplified DNA were deposited onto a 40 × 75 mm PicoTiterPlate equipped with an eight-lane gasket. This gasket divides the plate into eight identical regions (lanes) in which the pyrosequencing reactions occur during GS 20 sequencing runs. Each species was initially assigned four lanes on a single plate for a titration sequencing run, which is a standard preliminary sequencing run in which the relative quality of GS 20 libraries is assessed. Preliminary analyses of these data allowed for the estimation of the number of additional GS 20 sequencing lanes (three for *Nandina*, five for *Platanus*) on a second plate that were necessary to obtain approximately 20× coverage for each genome. This second sequencing run will be referred to as the supplementary run.

DNA sequence data from the titration and supplementary runs were combined in a single assembly for each species using version 1.0.52.06 of the GS 20 Newbler sequence assembly software. These data are referred to as the combined run data. The combined data contigs were then imported into DOGMA [42] to determine their approximate positions within the plastid genome. Based on this information, putatively adjacent contigs were examined in Sequencher 4.2 (GeneCodes Corp., Ann Arbor, MI, USA) in order to unite any contigs where short sequence overlap at the ends went undetected in the initial assembly. Gaps between contigs were bridged by designing custom primers near the ends of the GS 20 contigs for PCR and conventional capillary-based sequencing.

To estimate the accuracy of the GS 20 sequence, custom primers were designed to check all possible frame shift errors encountered in the preliminary DOGMA annotation of the GS 20 sequence of both genomes using PCR and conventional sequencing. In addition, the four junctions between the inverted repeat (IR) and single-copy (SC) regions of both genomes were sequenced conventionally, as was the entire IR region for both genomes. The IR regions were amplified using the recently described ASAP method [43], which utilizes a set of 27 overlapping primer pairs that are designed to obtain extensive coverage of the IR across the phylogenetic diversity of angiosperms. RCA product derived from the same chloroplast isolations used in GS 20 sequencing was utilized for all amplifications involving the single-copy regions of *Nandina *and for all regions of *Platanus*. The IR region of *Nandina *was amplified from a separate total DNA isolation from a different individual collected at Kanapaha Botanical Gardens in Gainesville, Florida (A. Dhingra s.n.).

The completed genome sequences were annotated using DOGMA and are available in GenBank.

## Authors' contributions

MJM isolated chloroplasts and performed RCA reactions, performed PCR for all regions outside of the IR, annotated the genomes, performed all error analyses, and drafted the manuscript. AD performed ASAP PCR for the IR regions. DES and PSS participated in the design of the study. RS performed laboratory protocols for GS 20 sequencing. WGF coordinated the GS 20 sequencing, assembled the raw data, and contributed to writing the Methods. KMF participated in the coordination of the study. All authors read and approved the final manuscript.

## Figures and Tables

**Table 1:** Characteristics of the GS 20 combined run data assemblies

	*Nandina*	*Platanus*
combined run data length	130503 bp	136335 bp
no. of combined data contigs	8	10
average contig length	16313 bp	13634 bp
size of largest contig	35901 bp	28803 bp
total no. of reads	31019	23743
average read length	103.6 bp	99.8 bp
overall average read depth (incl. one IR)	24.6×	17.3×
overall average read depth (incl. both IRs)	20.5×	14.6×
IR average read depth	24.2×	28.2×
SC average read depth	24.7×	14.9×
proportion of bases ≥ Q40	99.8%	99.4%
no. of gaps	9	11
total gap length	34 bp	390 bp
average gap length	3.8 bp	35.5 bp
no. of zero-length gaps	7	5
size of largest gap	32 bp	170 bp

Characteristics of the GS 20 combined run data assemblies. The overall average read depth is calculated in two ways: by including one copy of the inverted repeat (IR) region (to reflect the fact that the two copies of the IR are indistinguishable during genome sequencing, and are therefore contigged together) and by including both copies of the IR region. SC = single-copy region.

**Table 2:** Basic characteristics of the *Nandina *and *Platanus plastid *genomes

	*Nandina*	*Platanus*
total genome length	156599	161791
IR length	26062	25066
SSC length	19002	19509
LSC length	85473	92150
total length of coding sequence (both IRs)	92284	91397
total length of coding sequence (one IR)	75763	75716
total length of noncoding sequence (both IRs)	64315	70394
total length of noncoding sequence (one IR)	54774	61009
overall G/C content	38.3%	38.0%

Basic characteristics of the *Nandina *and *Platanus *plastid genomes. All lengths are given in base pairs (bp). IR = inverted repeat region; SSC = small single-copy region; LSC = large single-copy region.

**Table 3:** List of genes present in the plastid genomes of *Nandina *and *Platanus*

Gene Class				
Ribosomal RNAs	*rrn4.5 *(×2)	*rrn5 *(×2)	*rrn16 *(×2)	*rrn23 *(×2)
				
Transfer RNAs	*trnH-GUG*	trnK-UUU*	*trnQ-UUG*	*trnS-GCU*
	trnG-UCC*	*trnR-UCU*	*trnC-GCA*	*trnD-GUC*
	*trnY-GUA*	*trnE-UUC*	*trnT-GGU*	*trnS-UGA*
	*trnG-GCC*	*trnfM-CAU*	*trnS-GGA*	*trnT-UGU*
	trnL-UAA*	*trnF-GAA*	trnV-UAC*	*trnM-CAU*
	*trnW-CCA*	*trnP-UGG*	*trnI-CAU *(×2)	*trnL-CAA *(×2)
	*trnV-GAC *(×2)	trnI-GAU* (×2)	trnA-UGC* (×2)	*trnR-ACG *(×2)
	*trnN-GUU *(×2)	*trnL-UAG*		
				
Photosystem I	*psaA*	*psaB*	*psaC*	*psaI*
	*psaJ*			
				
Photosystem II	*psbA*	*psbB*	*psbC*	*psbD*
	*psbE*	*psbF*	*psbH*	*psbI*
	*psbJ*	*psbK*	*psbL*	*psbM*
	*psbN*	*psbT*	*psbZ*	
				
Cytochrome b6/f	*petA*	petB*	petD*	*petG*
	*petL*	*petN*		
				
ATP synthase	*atpA*	*atpB*	*atpE*	atpF*
	*atpH*	*atpI*		
				
NADH	ndhA*	ndhB* (×2)	*ndhC*	*ndhD*
dehydrogenase	*ndhE*	*ndhF*	*ndhG*	*ndhH*
	*ndhI*	*ndhJ*	*ndhK*	
				
Ribosomal proteins				
large subunit	rpl2* (×2)	*rpl14*	rpl16*	*rpl20*
	*rpl22*	*rpl23 *(× 2)	*rpl32*	*rpl33*
	*rpl36*			
				
small subunit	*rps2*	*rps3*	*rps4*	*rps7 *(×2)
	*rps8*	*rps11*	rps12* (×2)	*rps14*
	*rps15*	rps16*	*rps18*	*rps19*
				
RNA polymerase	*rpoA*	*rpoB*	rpoC1*	*rpoC2*
				
Miscellaneous	*accD*	*ccsA*	*cemA*	clpP*
proteins	*infA*	*matK*	*rbcL*	
				
Hypothetical	*ycf1*	*ycf2 *(×2)	ycf3*	*ycf4*
proteins	*ycf15 *(×2; present in *Nandina*; Ψ in *Platanus*)			

List of genes present in the plastid genomes of *Nandina *and *Platanus*. Genes with an asterisk (*) contain introns; genes that are present as duplicate copies due to their position within the inverted repeat regions are indicated as (×2). Ψ = pseudogene.

**Table 4:** Error rates for the GS 20 plastid genome sequence

Region	*Nandina*	*Platanus*	combined
overall genome	0.043	0.031	0.037
overall SC	0.030	0.064	0.047
overall IR	0.054	0.004	0.029
overall coding	0.027	0.029	0.028
overall noncoding	0.085	0.036	0.062
SC coding	0.036	0.055	0.046
SC noncoding	0.000	0.161	0.057
IR coding	0.018	0.000	0.009
IR noncoding	0.115	0.011	0.063

Observed error rates for the GS 20 plastid genome sequence of *Nandina*, *Platanus*, and both genomes combined (given in percent). These error rates are based on known GS 20 errors discovered in regions of conventional comparison sequence. Only one copy of the IR was included in error calculation.

**Table 5:** Raw values used in error calculations

	*Nandina*		*Platanus*		combined	
Region	# errors	length (bp)	# errors	length (bp)	# errors	length (bp)

overall genome	20	46134	14	45249	34	91383
overall SC	6	20072	13	20183	19	40255
overall IR	14	26062	1	25066	15	51128
overall coding	9	33170	10	34006	19	67176
overall noncoding	11	12946	4	11243	15	24189
SC coding	6	16649	10	18325	16	34974
SC noncoding	0	3405	3	1858	3	5263
IR coding	3	16521	0	15681	3	32202
IR noncoding	11	9541	1	9385	12	18926

Raw values that were used in calculations of observed error in GS 20 plastid genome sequence. Length refers to the length of conventional sequence data used in error calculations.

**Table 6:** Characteristics of GS 20 sequencing errors

	*Nandina*	*Platanus*	combined
proportion of length-variant HR errors	100.0	61.5	84.8
proportion of TLI HR errors	0.0	38.5	15.2
proportion of A/T HR errors	95.0	100.0	97.0
proportion of C/G HR errors	5.0	0.0	3.0
proportion of errors associated with HR sets	55.0	46.2	51.5
proportion of errors associated with HRs ≥ 5	45.0	76.9	57.6
average length of HR associated with error	5.4	6.5	5.8
proportion of HR-associated insertion errors	85.0	69.2	78.8
proportion of HR-associated deletion errors	15.0	30.8	21.2

Characteristics of observed GS 20 sequencing errors that were associated with homopolymer runs. All values are reported in percent. HR = homopolymer run; TLI = transposition-like insertion (see text).

**Table 7:** GS 20 quality scores associated with insertion errors

	# of insertion errors		
GS 20 quality scores	*Nandina*	*Platanus*	combined

< 20	14	8	22
20–40	2	1	3
> 40	1	1	2

Number of insertion errors in GS 20 combined sequence, as a function of the GS 20 phred-equivalent quality score at the insertion error site.

## References

[B1] Olmstead RG, Palmer JD (1994). Chloroplast DNA systematics: A review of methods and data analysis. Am J Bot.

[B2] Savolainen V, Chase MW (2003). A decade of progress in plant molecular phylogenetics. Trends Genet.

[B3] Bungard RA (2004). Photosynthetic evolution in parasitic plants: insight from the chloroplast genome. Bioessays.

[B4] Maliga P (2004). Plastid transformation in higher plants. Annu Rev Plant Biol.

[B5] Grevich JJ, Daniell H (2005). Chloroplast genetic engineering: Recent advances and future perspectives. Crit Rev Plant Sci.

[B6] Dhingra A, Daniell H, Salinas J, Sanchez-Serrano JJ (2005). Chloroplast genetic engineering via organogenesis or somatic embryogenesis. Arabidopsis Protocols.

[B7] Jansen RK, Raubeson LA, Boore JL, dePamphilis CW, Chumley TW, Haberle RC, Wyman SK, Alverson AJ, Peery R, Herman SJ, Fourcade HM, Kuehl JV, McNeal JR, Leebens-Mack J, Cui L (2005). Methods for obtaining and analyzing whole chloroplast genome sequences. Methods Enzymol.

[B8] Angiosperm Tree of Life project. http://www.flmnh.ufl.edu/angiospermATOL/.

[B9] Green Tree of Life project. http://ucjeps.berkeley.edu/TreeofLife.

[B10] Comparative chloroplast genomics project. http://evogen.jgi.doe.gov/second_levels/chloroplasts/jansen_project_home/chlorosite.html.

[B11] Metzker ML (2005). Emerging technologies in DNA sequencing. Genome Res.

[B12] Shendure J, Mitra RD, Varma C, Church GM (2004). Advanced sequencing technologies: methods and goals. Nat Rev Genet.

[B13] Ronaghi M, Uhlen M, Nyren P (1998). A sequencing method based on real-time pyrophosphate. Science.

[B14] Margulies M, Egholm M, Altman WE, Attiya S, Bader JS, Bemben LA, Berka J, Braverman MS, Chen YJ, Chen Z, Dewell SB, Du L, Fierro JM, Gomes XV, Godwin BC, He W, Helgesen S, Ho CH, Irzyk GP, Jando SC, Alenquer ML, Jarvie TP, Jirage KB, Kim JB, Knight JR, Lanza JR, Leamon JH, Lefkowitz SM, Lei M, Li J, Lohman KL, Lu H, Makhijani VB, McDade KE, McKenna MP, Myers EW, Nickerson E, Nobile JR, Plant R, Puc BP, Ronan MT, Roth GT, Sarkis GJ, Simons JF, Simpson JW, Srinivasan M, Tartaro KR, Tomasz A, Vogt KA, Volkmer GA, Wang SH, Wang Y, Weiner MP, Yu P, Begley RF, Rothberg JM (2005). Genome sequencing in microfabricated high-density picolitre reactors. Nature.

[B15] Ronaghi M (2001). Pyrosequencing sheds light on DNA sequencing. Genome Res.

[B16] Ewing B, Green P (1998). Base-calling of automated sequencer traces using phred. II. Error probabilities. Genome Res.

[B17] Meldrum D (2000). Automation for genomics, part one: preparation for sequencing. Genome Res.

[B18] Poinar HN, Schwarz C, Qi J, Shapiro B, Macphee RD, Buigues B, Tikhonov A, Huson DH, Tomsho LP, Auch A, Rampp M, Miller W, Schuster SC (2006). Metagenomics to paleogenomics: large-scale sequencing of mammoth DNA. Science.

[B19] Edwards RA, Rodriguez-Brito B, Wegley L, Haynes M, Breitbart M, Peterson DM, Saar MO, Alexander S, Alexander EC, Rohwer F (2006). Using pyrosequencing to shed light on deep mine microbial ecology under extreme hydrogeologic conditions. BMC Genomics.

[B20] Goldberg SM, Johnson J, Busam D, Feldblyum T, Ferriera S, Friedman R, Halpern A, Khouri H, Kravitz SA, Lauro FM, Li K, Rogers YH, Strausberg R, Sutton G, Tallon L, Thomas T, Venter E, Frazier M, Venter JC (2006). A Sanger/pyrosequencing hybrid approach for the generation of high-quality draft assemblies of marine microbial genomes. Proc Natl Acad Sci U S A.

[B21] Hofreuter D, Tsai J, Watson RO, Novik V, Altman B, Benitez M, Clark C, Perbost C, Jarvie T, Du L, Galan JE (2006). Unique features of a highly pathogenic Campylobacter jejuni strain. Infect Immun.

[B22] Sogin ML, Morrison HG, Huber JA, Welch DM, Huse SM, Neal PR, Arrieta JM, Herndl GJ (2006). Microbial diversity in the deep sea and the underexplored "rare biosphere". Proc Natl Acad Sci U S A.

[B23] Soltis DE, Soltis PS, Endress PK, Chase MW (2005). Phylogeny and Evolution of Angiosperms.

[B24] Anderson CL, Bremer K, Friis EM (2005). Dating phylogenetically basal eudicots using rbcL sequences and multiple fossil reference points. Am J Bot.

[B25] Palmer JD, Hermann RG (1991). Plastid chromosomes: structure and evolution. Cell Culture and Somatic Cell Genetics of Plants, vol 7A, The Molecular Biology of Plastids.

[B26] Raubeson LA, Jansen RK, Henry RJ (2005). Chloroplast genomes of plants. Plant Diversity and Evolution: Genotypic and Phenotypic Variation in Higher Plants.

[B27] Shinozaki K, Ohme M, Tanaka M, Wakasugi T, Hayashida N, Matsubayashi T, Zaita N, Chunwongse J, Obokata J, Yamaguchi-Shinozaki K, Ohto C, Torazawa K, Meng BY, Sugita M, Deno H, Kamogashira T, Yamada K, Kusuda J, Takaiwa F, Kato A, Tohdoh N, Shimada H, Sugiura M (1986). The complete nucleotide sequence of the tobacco chloroplast genome: its gene organization and expression. EMBO J.

[B28] Wakasugi T, Tsudzuki T, Sugiura M (2001). The genomics of land plant chloroplasts: Gene content and alteration of genomic information by RNA editing. Photosynth Res.

[B29] Schmitz-Linneweber C, Maier RM, Alcaraz JP, Cottet A, Herrmann RG, Mache R (2001). The plastid chromosome of spinach (Spinacia oleracea): complete nucleotide sequence and gene organization. Plant Mol Biol.

[B30] Steane DA (2005). Complete Nucleotide Sequence of the Chloroplast Genome from the Tasmanian Blue Gum, Eucalyptus globulus (Myrtaceae). DNA Res.

[B31] Tsudzuki T, Wakasugi T, Sugiura M (2001). Comparative analysis of RNA editing sites in higher plant chloroplasts. J Mol Evol.

[B32] Rogers YH, Venter JC (2005). Genomics: massively parallel sequencing. Nature.

[B33] Powell W, Morgante M, Andre C, McNicol JW, Machray GC, Doyle JJ, Tingey SV, Rafalski JA (1995). Hypervariable microsatellites provide a general source of polymorphic DNA markers for the chloroplast genome. Curr Biol.

[B34] Lasken RS, Egholm M (2003). Whole genome amplification: abundant supplies of DNA from precious samples or clinical specimens. Trends Biotechnol.

[B35] Strauss BS (1999). Frameshift mutation, microsatellites and mismatch repair. Mutat Res.

[B36] Provan J, Powell W, Hollingsworth PM (2001). Chloroplast microsatellites: new tools for studies in plant ecology and evolution. Trends Ecol Evol.

[B37] Zirvi M, Nakayama T, Newman G, McCaffrey T, Paty P, Barany F (1999). Ligase-based detection of mononucleotide repeat sequences. Nucleic Acids Res.

[B38] Clarke LA, Rebelo CS, Goncalves J, Boavida MG, Jordan P (2001). PCR amplification introduces errors into mononucleotide and dinucleotide repeat sequences. Mol Pathol.

[B39] Liepelt S, Kuhlenkamp V, Anzidei M, Vendramin GG, Ziegenhagen B (2001). Pitfalls in determining size homoplasy of microsatellite loci. Mol Ecol Notes.

[B40] Cosner ME, Jansen RK, Palmer JD, Downie SR (1997). The highly rearranged chloroplast genome of Trachelium caeruleum (Campanulaceae): multiple inversions, inverted repeat expansion and contraction, transposition, insertions/deletions, and several repeat families. Curr Genet.

[B41] Dean FB, Nelson JR, Giesler TL, Lasken RS (2001). Rapid amplification of plasmid and phage DNA using Phi 29 DNA polymerase and multiply-primed rolling circle amplification. Genome Res.

[B42] Wyman SK, Jansen RK, Boore JL (2004). Automatic annotation of organellar genomes with DOGMA. Bioinformatics.

[B43] Dhingra A, Folta KM (2005). ASAP: amplification, sequencing & annotation of plastomes. BMC Genomics.

